# Opinion Exploiting genomics to improve the benefits of wheat: Prospects and limitations

**DOI:** 10.1016/j.jcs.2022.103444

**Published:** 2022-05

**Authors:** Peter R. Shewry, Alison Lovegrove, Luzie U. Wingen, Simon Griffiths

**Affiliations:** a Rothamsted Research, Harpenden, Hertfordshire, AL5 2JQ, UK; b John Innes Centre, Norwich Research Park, Colney Lane, Norwich, NR4 7UH, UK

**Keywords:** Wheat, Biotechnology, Health, Genomics, Molecular breeding

## Abstract

• The last decade has seen an explosion in wheat genomic resources.
• These include genomic sequences and high-density molecular markers.
• Genomic resources allow the exploitation of novel genetic variation.
• Genomic resources are being used to improve the health benefits of wheat.

## Graphical abstract

**Figure:**
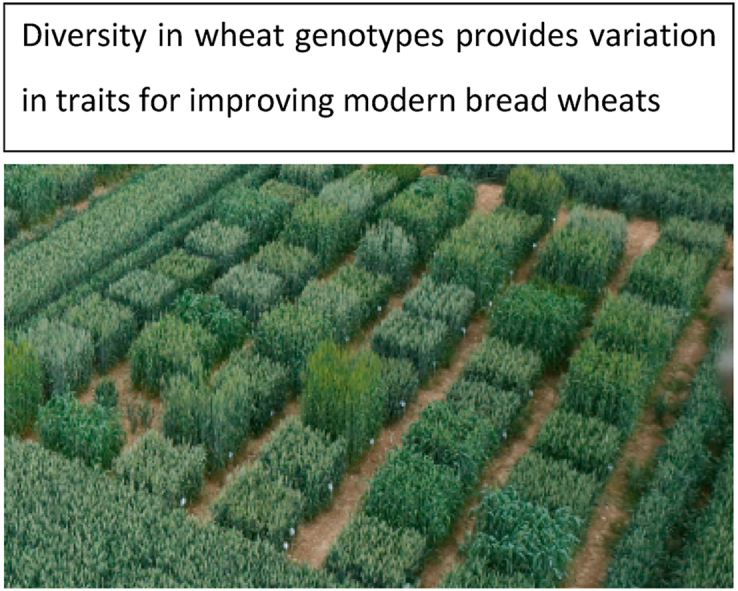

## 1 Background

Conventional breeding has been immensely successful in increasing crop production to meet the demands of the growing global population, particularly for wheat where production has increased by over three-fold over the last 60 years without a significant increase in the area of land used. However, the pace of improvement by conventional breeding is slow and limited by the range of variation present in wheat and species with which it can be crossed. Genomics can be defined as “an interdisciplinary field of biology focusing on the structure, function, evolution, mapping, and editing of genomes” (Wikipedia). As such it has the potential to revolutionise crop improvement, by accelerating the rate of progress and increasing the range of variation that is available. Despite this potential, progress in the application of biotechnology to improve wheat has been slow, particularly when applied to the quality of the grain for processing and nutrition. We will therefore consider the reasons for this and identify priorities for future research.

## 2 Wheat genomic resources

### 2.1 Genomic sequences

There is no doubt that the slow development of genomic resources for wheat has significantly affected the application of genomics. For example, whereas assembled genome sequences for rice and maize were reported in 2002 and 2009, the genome assembly for the reference wheat genotype Chinese Spring was not reported until 2018 (International Wheat Genetics Sequencing Consortium, 2018). This slow progress resulted from several factors, including technical challenges associated with the large genome size (16 Gb compared to 400–430 Mb for rice and 2.3–2.7 Gb for maize), the polyploid nature and high content (>80%) of repetitive DNA. However, it also reflected limited investment by multinational plant biotechnology companies who focused on hybrid crops with larger profit margins. Nevertheless, progress has since been rapid, with increasing numbers of genotypes being sequenced (Walkowiak et al., 2020). Impressive progress has also been made in generating other genomic resources, including high density molecular marker systems which allow the detection of up to 1 million single nucleotide polymorphisms (SNPs) in a single analysis (Winfield et al., 2016) and the sequencing of 10 million induced mutations in bread and durum wheats (Krasileva et al., 2017). The latter allows researchers to determine gene function by analysing lines with specific mutations.

The primary germplasm resources are vast, for example, the FAO reports more than 850,000 accessions of *Triticum* in more than 200 genebanks. This natural diversity is increasingly accessible because of publicly available gene discovery resources such as segregating populations for crosses with landraces (Wingen et al., 2017), wild relatives (King et al., 2017), and modern cultivars (Gardner et al., 2016). Indeed, the rate of publication of genetic resources is slow compared with the rate of deposition in genebanks which can be accessed using online browsers (eg. https://www.seedstor.ac.uk/search-browsecollections.php). Many genebank collections are now accompanied by high density genotyping data allowing the identification of introgressions (Przewieslik-Allen et al., 2012), or even fully sequenced genomes (Gaurav et al. 2012). Precise germplasm stocks such as near isogenic lines also provide powerful tools for the detailed characterisation of single loci (Farré et al., 2016).

The global interest in the exploitation of wheat genomics is demonstrated by the fact that it is the most accessed species in the Ensembl Plants portal (http://plants.ensembl.org), above rice and Arabidopsis.

### 2.2 Development and exploitation of marker systems

The most successful application of genomics to wheat improvement has been in the development and application of molecular markers for breeding (“molecular breeding”). The identification of polymorphisms in DNA sequences within, or closely linked to, genes allows rapid selection and has been used in conventional breeding (based on Mendelian genetics) to select for traits which are difficult or expensive to identify for using conventional morphological or biochemical screens, and for traits which show continuous variation resulting from the effects of multiple genes (quantitative trait loci, QTLs). For example, QTLs have been identified for dietary fibre content (Lovegrove et al., 2020) and molecular markers developed to introgress the trait into varieties and track it through breeding pedigrees.

Markers are also widely used in genome-wide association studies (GWAS) which allow the identification of loci which determine individual traits and in genomic selection (GS), where they are used as predictors of performance, rather than to identify and follow single loci. GS can therefore be used to improve traits controlled by many genes with minor effects (Guzman et al., 2016) and, while it is still extremely challenging for complex traits such as yield (Juliana et al., 2020), is now widely used in commercial wheat breeding programmes.

## 3 Use of genomics to increase variation in traits

Crop genetic improvement depends on exploiting genetic variation from two sources. Firstly, the reassortment of genes during meiosis generates a wider range of variation than is present in the parental genotypes: this is called “transgressive segregation”. Secondly, variation may be introduced from specific genotypes, which may be cultivars from different geographical regions, older types grown in historical times (including landraces), other wheat species and subspecies (including emmer, einkorn and spelt) and related wild grasses.

Although bread wheat is a relatively young crop, having originated about 10,000 years ago, it is immensely diverse. This diversity results partly from gene flow between bread wheat and cultivated and wild forms of tetraploid wheat, particularly wild emmer (Przewieslik-Allen et al., 2012), but also from genome plasticity, both of which are facilitated by the hexaploid nature. As discussed above, over 80% of the genome is non-coding repetitive DNA, including mobile elements, which facilitates the generation of variation by gene duplication and deletion and by insertion into regulatory and coding sequences. However, wheat breeders exploit only a small proportion of the available diversity, and there is evidence of reduced diversity in modern bread wheat cultivars compared to landraces and older genotypes from the same geographical area (Lovegrove et al., 2020).

Current research focuses on exploiting variation from two main sources. Firstly, landrace collections provide a “snapshot” of the diversity present when they were grown. For example, the Watkins collection of landraces was collected in 34 countries in the 1930s (Wingen et al., 2017). About 1050 genotypes are available of which over 800 have been sequenced. These sequences, combined with high density marker analyses of crosses with modern wheats, allow the identification of genomic regions which can be introgressed into modern cultivars to widen the range of variation. Secondly, libraries of mutant lines are being used to confirm the identity of genes for key traits and to identify mutants, and mutant combinations, which result in novel phenotypes.

## 4 Application of genomics to improving nutritional and health benefits

Despite the extensive genomic resources there has been limited impact on the quality of wheat for human health. One reason for this is that there is little demand for improved wheat products from consumers, processors and health professionals, with the notable exception of mineral micronutrients. Consequently, the level of interest from wheat breeders has also been low: wheat breeding is a lengthy (5–7 years from first crosses to cultivars) and expensive process and therefore focuses on traits with well established impacts on yield and processing quality. However, there are also technical challenges due to our limited knowledge of the pathways and mechanisms determining the traits.

### 4.1 Mineral micronutrients

Deficiencies in iron (Fe) and zinc (Zn) affect 2 billion people globally, particularly in the developing world. Cereals are major sources of minerals in the diet and hence have received considerable attention, particularly by CIMMYT and other CGIAR research centres as part of the HarvestPlus programme. The amounts of Fe and Zn in wheat grain are strongly affected by the available minerals in soils, but in broad terms both range from about 20 to 50 mg/kg DM.

There are two factors to consider in improving micronutrient content. Firstly, it is necessary to increase the total contents in the grain. However, minerals are concentrated in the aleurone and embryo tissues, largely as phytates which have low solubility and hence low bioavailability (about 10% of total grain Fe and 25% of zinc). It is therefore also important to increase their concentrations in the starchy endosperm (the source of white flour) and their bioavailability. Velu et al. (2018) used GWAS to identify two QTL regions for high grain zinc and developed lines with about 40% increases compared with control lines. Lines adapted for South Asia are being used to study the effects on the zinc status of consumers.

The increase in grain minerals requires greater transport of the minerals into the grain, and genomic resources can be exploited to identify transcription factors which may affect the uptake of minerals by the plant and partitioning into the developing grain (Uauy et al., 2006; Ali and Borrill, 2020). However, these increases are not associated with changes in mineral form or location and novel strategies are required to achieve these.

Transgenesis has been used to increase the concentrations of bioavailable minerals in the starchy endosperm tissue (Balk et al., 2018). Minerals enter the developing grain from the vascular bundle in the crease and are then transported across the starchy endosperm cells to the sink tissues (embryo and aleurone). Accumulation in the starchy endosperm therefore results from the disruption of this pathway, which has been achieved by the expression of transgenes encoding proteins which transport the minerals into the vacuoles of the starchy endosperm cells or synthesise a naturally occurring metal chelator (nicotianamine). However, transgenic approaches are not acceptable in many countries and a more detailed understanding of the genes and pathways involved in mineral transport is required to facilitate the use of mutagenesis or gene editing to effect similar changes.

### 4.2 Grain carbohydrates: starch and fibre

Reducing the rates of starch digestion and glucose release in the human gastrointestinal tract is a key target to reduce the risk of type 2 diabetes. The rate of starch digestion is affected by starch composition, particularly the proportion of amylose, and high amylose lines with lower digestibility have been produced by transgenesis and mutagenesis (Hazard et al., 2020). However, changes in starch composition pose a challenge for processors as they affect the functional properties while associated adverse effects on yield affect cost.

The content of dietary fibre also affects the rate of starch digestion, possibly by regulating the rate of food breakdown and the accessibility of the starch granules to enzymes, and fibre has a wide range of additional benefits. The content of dietary fibre in white flour varies by up to two-fold and this can be exploited to increase grain fibre content (Lovegrove et al., 2020).

However, more detailed understanding of the regulation of synthesis of both starch and dietary fibre components should allow the more precise restructuring of grain composition.

### 4.3 Vitamins and phytochemicals

Wheat is an important sources of vitamins, notably B vitamins (thiamine (B1), riboflavin (B2), niacin (B3), pyridoxine (B6) and folates (B9)), and of phytochemicals with putative health benefits, notably ferulic acid and other phenolic acids. These compounds are largely concentrated in the bran and hence increases in white flour would be beneficial. They are synthesised by complex multi-step biochemical pathways and transgenesis has been used to increase the activities of rate-limiting enzymes in other plants (reviewed by Martin and Li, 2017). However, the success of this approach varies and a more promising approach is to manipulate the expression of transcription factors which regulate the biosynthetic pathways as reported for polyphenols in tomato (Martin and Li, 2017).

## 5 Conclusions

A wide range of genomic resources are available for wheat. Furthermore, the use of classical genetics, transgenesis and mutagenesis have shown that improvements in traits affecting health benefits can be achieved. However, long term improvements require subtle alterations in the compositions, locations and forms of components as well as amounts. These require a deeper understanding of the controls operating in the developing seed as well as non-transgenic approaches to develop genotypes for human consumption.

## Declaration of competing interest

The authors declare that they have no known competing financial interests or personal relationships that could have influenced the work reported in this paper.
